# Do nucleic acids moonlight as molecular chaperones?

**DOI:** 10.1093/nar/gkw291

**Published:** 2016-04-21

**Authors:** Brianne E. Docter, Scott Horowitz, Michael J. Gray, Ursula Jakob, James C.A. Bardwell

**Affiliations:** 1 Cellular and Molecular Biology Program, University of Michigan, Ann Arbor, MI 48109, USA; 2 Department of Molecular, Cellular, and Developmental Biology, University of Michigan, Ann Arbor, MI 48109, USA; 3 Howard Hughes Medical Institute, University of Michigan, Ann Arbor, MI 48109, USA

## Abstract

Organisms use molecular chaperones to combat the unfolding and aggregation of proteins. While protein chaperones have been widely studied, here we demonstrate that DNA and RNA exhibit potent chaperone activity *in vitro*. Nucleic acids suppress the aggregation of classic chaperone substrates up to 300-fold more effectively than the protein chaperone GroEL. Additionally, RNA cooperates with the DnaK chaperone system to refold purified luciferase. Our findings reveal a possible new role for nucleic acids within the cell: that nucleic acids directly participate in maintaining proteostasis by preventing protein aggregation.

## INTRODUCTION

One of the major impacts of cellular stress is the unfolding and aggregation of proteins. To deal with this problem, cells mobilize molecular chaperones, which bind to misfolded proteins and prevent their aggregation. Proteins are the most widely studied molecular chaperones, although osmolytes have also been shown to be active in preventing aggregation and in enhancing protein refolding (1,2). Recently, we reported that polyphosphate, a polymer of phosphate molecules, prevents protein aggregation both *in vitro* and *in vivo* (3). DNA, RNA and polyanions, including heparin, have been shown to accelerate the folding of a variety of nucleic acid binding proteins up to 30-fold (4). This acceleration is likely in part due to the stabilizing influence of specific binding (5).

Additionally, ribosomes have long been known to be involved in protein folding (6). Curiously, the naked V domain of 23S rRNA from multiple species is active in the refolding of a wide variety of proteins (7–10). Higher refolding yields are achieved for carbonic anhydrase, lactate dehydrogenase, malate dehydrogenase and lysozyme in the presence of the V domain of 23S rRNA, and this refolding activity is inhibited by antibiotics such as blasticidin that bind to the V domain (9,11). Mitochondrial 12S and 16S rRNA can also assist in the refolding of heat denatured EcoRI, luciferase and malate dehydrogenase (12).

The chaperone-like activities observed for ribosomal RNA, and the chemical similarity between the recently discovered chaperone polyphosphate (3) and nucleic acids, led us to wonder if RNA and DNA are generally active as chaperones. Here, we show that a wide variety of DNA and RNA species, including oligonucleotides as short as 19 bases, function as extremely efficient chaperones *in vitro*. They suppress the aggregation of the classic chaperone substrate citrate synthase (CS) over 400-fold more efficiently than does polyphosphate, and they are up to 300-fold more effective at preventing protein aggregation than the chaperone GroEL by weight (13,14). Furthermore, RNA can cooperate with the DnaK system to promote protein refolding. Their high concentration *in vivo* and their remarkable chaperone activity suggest that nucleic acids could play a vital role in maintaining the stability of the proteome.

## MATERIALS AND METHODS

### Nucleic acids and nucleotides

A summary of the nucleic acids used in this study can be found in Table 1. Genomic dsDNA, the sodium salt of DNA from herring testes (42% GC, Sigma-Aldrich) was purified with a phenol-chloroform extraction and ethanol precipitation as described previously (15); the final A_260_/A_280_ was 1.8–1.9. The salt concentration in control experiments was chosen to be 1.5 times the concentration of DNA base pairs (bp), based on work by Manning, who found that 1.5 sodium ions bound per bp (16). DNA fragmentation was performed using sonication for indicated periods of time on ice. RNA (from torula yeast, Sigma-Aldrich) was dissolved in water just before use (A_260_/A_280_ = 2.0-2.1). An equimolar dNTP mix (Promega) was used as the dNTP control. 2′-deoxycytidine-5′-monophoshate, 2′-deoxyguanosine-5′-monophosphate, 2′-deoxyadenosine-5′-monophosphate and thymidine-5′-monophosphate (Sigma-Aldrich) were mixed in water at a 1:1:1:1 molar ratio and used as the dNMP control. L-proline, D-sorbitol and D-sucrose were purchased from MP Biomedicals, Sigma-Aldrich and Fisher Scientific, respectively. Desalted DNA and RNA oligonucleotides were commercially synthesized (Integrated DNA Technologies). DNA oligos were resuspended to 100 μM in 10 mM Tris, 1 mM EDTA, pH 8. RNA oligos were resuspended to 300 uM in nuclease-free water. Sequences used in Figure 2A were 5′- TCGTTTTACCGCACCCCA-3′ (18 bases) and 5′-TAGCCGCTATTTTTTTGTCCTGAATGATGTTTGACACTACCGAGGTGTACTGTGTAGGCTGGAGCTGCTTC-3′ (71 bases). DNA homopolymers were 20 bases in length, and RNA homopolymers were 19 bases in length. Oligos of random sequence (Figure 2B, Supplementary Figure S5) were machine-made random at all bases for the indicated length. For strandedness experiments (Figure 4D), complimentary oligos with sequences of 5′- TGGGGTGCGGTAAAACGA-3′ (oligo A) and 5′- TCGTTTTACCGCACCCCA-3′ (oligo B) were used. Annealing of the oligos was performed by heating an equimolar mixture of the two oligos to 95°C and slowly reducing the temperature to 4°C.

**Table 1.** Nucleic acids used in this study

Name	Details	Used in Figures
Genomic dsDNA	Genomic DNA sodium salt from herring testes	Figures 1, 3, 4A, 4B, 4E, 5E, Figures S1, S2, S3, S4, S6
Bulk RNA	Total RNA from Torula yeast	Figure 5E, Figure S3
dNTPs	Equimolar dNTP mix	Figure 2A
dNMPs	Equimolar dNMP mix	Figure 2A
DNA oligos	Synthesized single stranded oligos resuspended in TE	
poly(dA)	5′-AAAAAAAAAAAAAAAAAAAA-3′	Figure 4C
poly(dC)	5′-CCCCCCCCCCCCCCCCCCCC-3′	Figure 4C
poly(dG)	5′-GGGGGGGGGGGGGGGGGGGG-3′	Figure 4C
poly(dT)	5′-TTTTTTTTTTTTTTTTTTTT-3′	Figure 4C
18 base ssDNA	5′-TCGTTTTACCGCACCCCA-3′	Figure 2A
71 base ssDNA	5′-TAGCCGCTATTTTTTTGTCCTGAATGATGTTTGACACTACCGAGGTGTACTGTGTAGGCTGGAGCTGCTTC-3′	Figure 2A
Random	Oligos were machine-made random at all bases, at lengths of 15, 30, 45 or 60 bases	Figure 2B, Figure S5
ssDNA A	5′- TGGGGTGCGGTAAAACGA-3′	Figure 4D
ssDNA B	5′- TCGTTTTACCGCACCCCA-3′	Figure 4D
RNA oligos	Synthesized and resuspended in nuclease-free water	
poly(A)	5′-AAAAAAAAAAAAAAAAAAA-3′	Figures 2C, 5B, 5F
poly(C)	5′-CCCCCCCCCCCCCCCCCCC-3′	Figure 5D, 5F
poly(G)	5′-GGGGGGGGGGGGGGGGGGG-3′	Figure 5C, 5F
poly(U)	5′-UUUUUUUUUUUUUUUUUUU-3′	Figures 5A, 5F, 6

### Thermal and chemical aggregation assays

**Figure 1.**
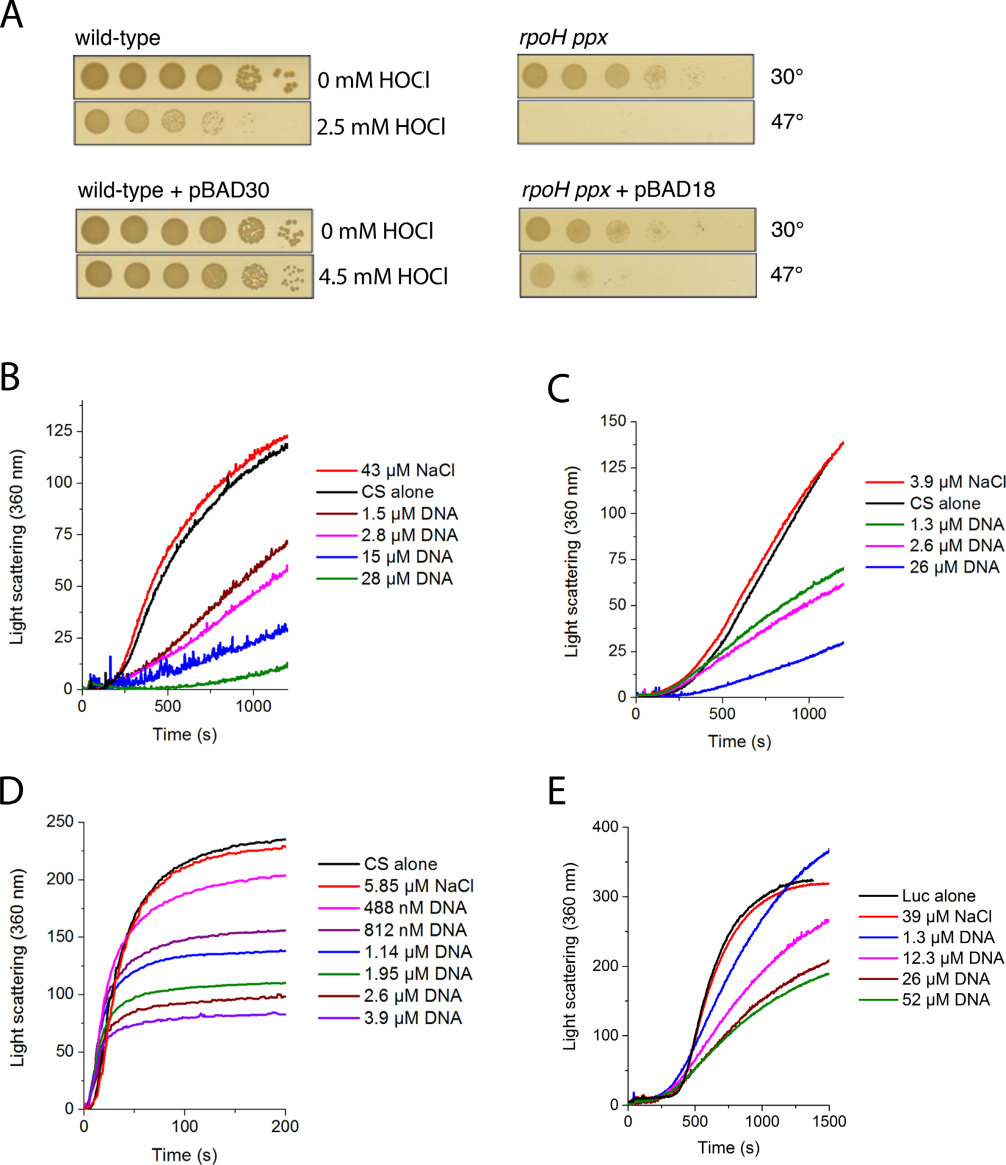
(**A**) Spot titers showing the *in vivo* response to oxidative stress (hypochlorous acid, HOCl) (left) and heat stress (right) in *E. coli* in the absence (top) and presence (bottom) of a plasmid. pBAD18 is a pBR322 origin of replication plasmid and thus has a copy number of 15–20 copies per cell. Since the *E coli* chromosome is 4639 kb and pBAD18 is 4.6 kb the proportion of DNA that comes from pBAD18 will be, at most, 2% of the cell DNA. Additionally, pBAD30, with an even lower copy number of 10–12 copies per cell, amounts to 1.2% of the cell DNA at most. (**B–E**) Protein aggregation assays as measured by light scattering. Genomic herring DNA was used; concentration is per basepair. Thermal denaturation of 150 nM citrate synthase (CS) in (**B**) HEPES buffer and (**C**) phosphate buffer at 41°C. (**D**) Chemical denaturation of 75 nM CS in HEPES buffer at 30°C. (**E**) Thermal denaturation of 140 nM luciferase in MOPS buffer at 40°C.

CS (from porcine heart, Sigma-Aldrich) at 150 nM was incubated at 41°C in 40 mM HEPES-KOH (except for Figure 1C, which used 10 mM potassium phosphate as buffer), pH 7.5, with constant stirring. QuantiLum Recombinant Luciferase (Promega) at 140 nM was incubated at 40°C in 40 mM MOPS, 50 mM KCl, pH 7.4, with constant stirring. Rhodanese (type II from bovine liver, Sigma-Aldrich) at 1.5 μM was incubated at 40°C in 40 mM potassium phosphate, pH 7.5, with constant stirring. Aggregation of 50 μM α-lactalbumin (from bovine milk, Sigma-Aldrich) in 50 mM sodium phosphate, 100 mM potassium chloride, 18 mM DTT, pH 7.0 was measured in a plate reader at 37°C, with absorbance at 360 nm measurements taken every 3 min with 10 s of shaking before each measurement. For chemically induced aggregation, CS at 12 μM was denatured in 6 M guanidine-HCl, 40 mM HEPES, for 15 h at 23°C, then diluted to 75 nM into 40 mM HEPES-KOH, pH 7.5, with constant stirring at 30°C. Light scattering (360 nm) at 90° was measured with an F-4500 Fluorescence Spectrometer (Hitachi). Experiments were repeated two or three times; representative curves are shown. The results were reproducible, showing similar dose-dependent curves when experiments were carried out on different days. For experiments with homopolymeric RNA, the RNase inhibitor RNasin Plus (Promega) was added at a final concentration of 80 U/ml to all reactions, including the non-RNA controls.

### Luciferase spin down assays

Around 3.3 μM luciferase was incubated in 10 mM potassium phosphate, pH 7.5 in 100 μl reactions at varying temperatures for 15 min, then centrifuged at 16 100 × *g* for 20 min at 4°C. The supernatant (∼97 μl) and pellet were separated, and the pellet was immediately dissolved in denaturing SDS loading buffer. Ten percent of the supernatant volume and 10% of the dissolved pellet volume were run on an SDS-PAGE gel, which was visualized with coomassie blue. Experiments were performed twice, and representative gels are shown. For experiments testing the effect of DNase, all reactions additionally contained 1 mM MgCl_2_ and 50 μM CaCl_2_. For reactions containing DNase, 13.8 nM DNaseI (Sigma-Aldrich) was added, having been resuspended to 2 mg/ml in water before use.

### Efficiency calculations

To compare the efficiency of chaperone activity of nucleic acids with that of other chaperones, published data of aggregation assays with protein chaperones were compared with our aggregation assay data (13,17–19). Chaperone assays were chosen that had similar concentrations of substrate to our experiment, and that were done under thermal denaturation conditions. Concentrations of protein chaperone that achieved a similar level of aggregation reduction as our nucleic acids were compared to one another on a mass-to-mass basis in order to determine the fold level of activity. We always erred on the side of ascribing less activity to the nucleic acids in order to account for differences between assays. For example, the activity of polyA RNA oligos (285 nM bases) appeared equivalent to 74 nM GroEL, and 570 nM bases was significantly stronger than 74 nM GroEL as reported by Höll-Neugebauer *et al*. (13); so for the calculation, we compared the 74 nM GroEL to 570 nM nucleotide bases in the calculation of mass equivalence. The 74 nM GroEL is 305 times heavier than the 570 nM bases, leading to our statement that the nucleic acids are at least 300-fold more efficient. The mass of the nucleic acids was estimated to be 330 Da per DNA base and 340 Da per RNA base. GroEL is 800 kDa, bovine Hsp90 is 84 kDa, *Escherichia coli* DnaK is 69 kDa, *E. coli* Hsp33 is 33 kDa, sucrose is 342 Da and polyphosphate is 79 Da per phosphate equivalent.

### Bacterial stress response assays

Hypochlorous acid (HOCl) stress response assays were performed as previously described (20). Wild-type *E. coli* MG1655 (21) containing either no plasmid or the medium-copy vector pBAD30 (22) were grown to mid-log phase in MOPS minimal glucose medium (Teknova) without or with 290 μM ampicillin, then incubated for 30 min with the indicated concentrations of HOCl. Heat shock assays were carried out with the strain MJG513 (MC4100 *rpoH*::*kan ppx*::*cat*) (3), containing either no plasmid or the high-copy vector pBAD18 (22). The *rpoH* gene encodes the heat shock sigma factor; *rpoH* null mutants exhibit a defective heat shock response, show reduced levels of chaperone proteins at high temperatures and are thus very temperature sensitive. These *rpoH* strains were grown to mid-log phase at their permissive temperature (30°C) in LB without or with 290 μM ampicillin, then incubated for 30 min at 47°C. After these stress treatments, cells were serially diluted and spot-titered on LB agar, then incubated overnight at 37°C for the bleach experiment or 30°C for the *rpoH* experiment.

### Circular dichroism (CD) experiments

CD spectra of herring genomic DNA (180 μM on a bp basis) and luciferase (3.3 μM on a monomer basis) were measured as a function of temperature both together and separately. The buffer was 10 mM potassium phosphate, pH 7.5. The temperature was ramped up from 15°C to 80°C at 1°C/min, pausing for CD measurements at every 5°C. CD spectra were measured from 340 to 195 nm. For analysis, the DNA alone CD spectrum was subtracted from the DNA plus luciferase spectrum at each temperature, and analyzed using BESTSEL (23).

### Luciferase refolding experiments

DnaK, DnaJ and GrpE were expressed and purified as previously described (24,25). Before inactivation, samples for 100% luciferase activity were removed and measured as described below. The scheme for luciferase refolding was similar to previously described protocols (3,24,26). All samples containing RNA were supplemented with RNasin Plus RNase inhibitors at 80 U/ml for the duration of the experiment. Nucleic acid and luciferase samples containing 150 nM luciferase were incubated together in glass vials at high temperature (40–42°C) for 20 min to inactivate luciferase (buffer was 50 mM MOPS, 50 mM KCl, pH 7.5). Triplicate inactivated samples were then pipetted into a glass 96-well plate for luciferase refolding at 25°C, with 300 rpm shaking, in 40 mM MOPS pH 7.5, 50 mM KCl, 0.1 mg/ml bovine serum albumin, 2 mM magnesium ATP and 2 mM DTT. DnaK, GrpE and DnaJ were added at 0.75, 0.75 and 0.15 μM, respectively to samples for refolding with the KJE system at the onset of refolding. At each time point, samples of the refolding mixture were diluted 1:20 in luciferase activity measurement buffer (100 mM potassium phosphate, 0.2 mM EDTA, 25 mM glycylglycine, 2 mM magnesium ATP, 0.5 mg/mL BSA and 70 μM luciferin, pH 7.5). Luminescence was measured in a FLUOstar Omega plate reader (BMG Labtech).

## RESULTS

While conducting experiments on bacterial stress resistance, we noticed that control strains containing nonexpressing DNA plasmids were more resistant to proteotoxic stress (e.g., bleach, heat treatment) than the same strains that lacked plasmids (Figure 1A). Given these observations, the previously observed chaperone-like properties of ribosomal RNA, and the consideration that nucleic acids are chemically similar to polyphosphate, we decided to test whether nucleic acids might generally act as chaperones.

**Figure 2.**
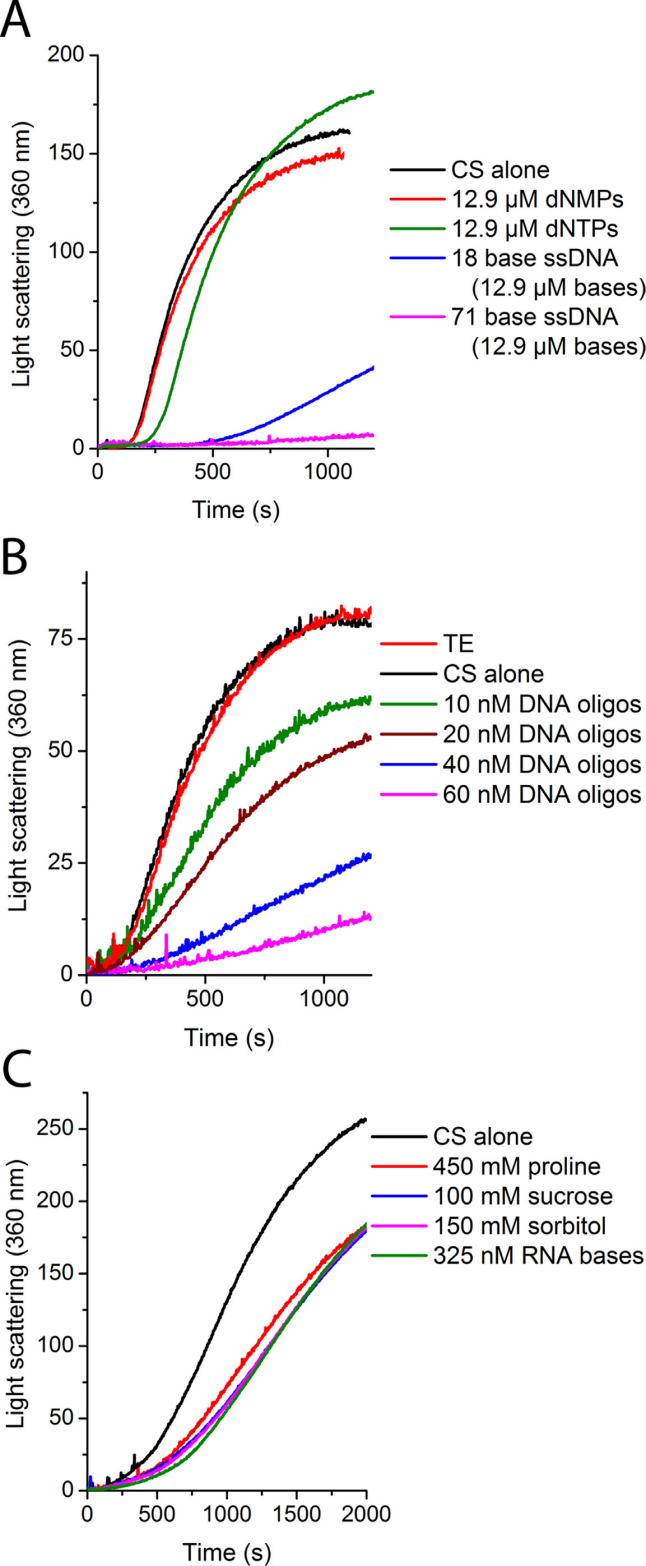
Thermal denaturation of 150 nM CS in HEPES buffer at 41°C. (**A**) Synthesized oligos 18 and 71 bases long of defined sequence were used (see ‘Materials and Methods’). (**B**) Synthesized ssDNA oligos of 30 bases long that were a mix of random sequences were used; concentration is per oligo. (**C**) Osmolytes or RNA were added at indicated concentrations. RNA was 19 base poly(A) oligos; concentration is in bases. For comparison of RNA and DNA chaperone effectiveness, please see Figure 5.

**Figure 3.**
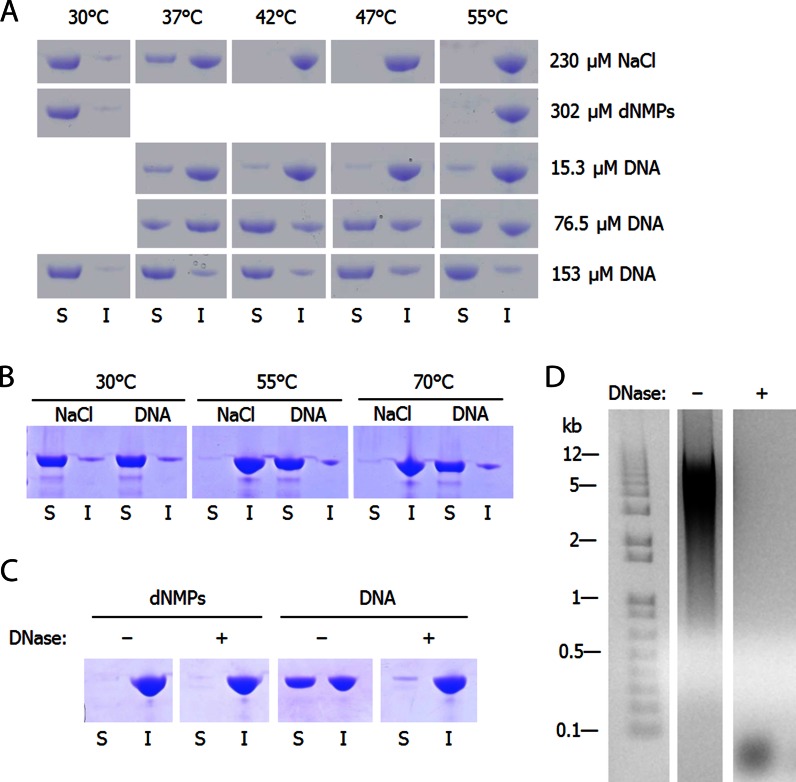
Spin-down experiments comparing soluble (S) and insoluble (I) purified luciferase after thermal denaturation. (**A** and **B**) 3.3 μM luciferase in phosphate buffer with 15 min heat shock at indicated temperatures; (**A**) DNA concentration is per basepair; (**B**) [DNA] = 180 μM basepairs; [NaCl] = 270 μM. (**C**) 3.3 μM luciferase in phosphate buffer plus 1 mM MgCl_2_ and 50 μM CaCl_2_ with 15 min heat shock at 42°C. [dNMPs] = 255 μM; [DNA] = 128 μM basepairs; [DNase] = 13.8 nM. (**D**) One percent agarose gel of DNA-containing soluble samples from panel **C**, where DNase was absent or present.

To determine whether nucleic acids possess chaperone attributes, we first tested the chaperone activity of purified herring genomic dsDNA by measuring its ability to prevent the aggregation of CS and luciferase. To our knowledge, neither of these proteins binds nucleic acids in the native state. We observed that with 150 nM CS, as little as 1.5 μM dsDNA (10 bp dsDNA per CS monomer) reduced the rate of CS aggregation under thermal stress by about half (Figure 1B). The chaperone activity did not depend on the buffer, as the activity persisted in both HEPES (Figure 1B) and 10 mM phosphate buffer (Figure 1C), as well as other buffers (Supplementary Figures S1 and S2). dsDNA displayed similar activity upon CS that had been denatured in 6 M guanidinium HCl (Figure 1D). Single nucleotides (dNMPs and dNTPs), however, had little to no effect on the rate of aggregation (Figure 2A), even at 8-fold higher concentrations than the 1.5 μM of nucleotide bases needed to reduce CS aggregation by 50% (Figures 1B and 2A). The potent chaperone activity of dsDNA was also observed against the thermally-induced aggregation of luciferase in both light scattering and spin-down experiments (Figures 1E and 3). In the spin-down assay, luciferase alone becomes completely insoluble at temperatures of 42°C and above, but addition of 55 bp DNA per luciferase molecule keeps the majority of luciferase soluble at temperatures as high as 70°C (Figure 3A and B). Overall, DNA served as an extremely effective anti-aggregation chaperone against chemically and thermally denatured CS and thermally denatured luciferase. However, like many other chaperones (27,28), DNA displays clear substrate specificity *in vitro*, as no significant activity was observed against rhodanese (Supplementary Figure S3), α-lactabumin (Supplementary Figure S4) or malate dehydrogenase.

**Figure 4.**
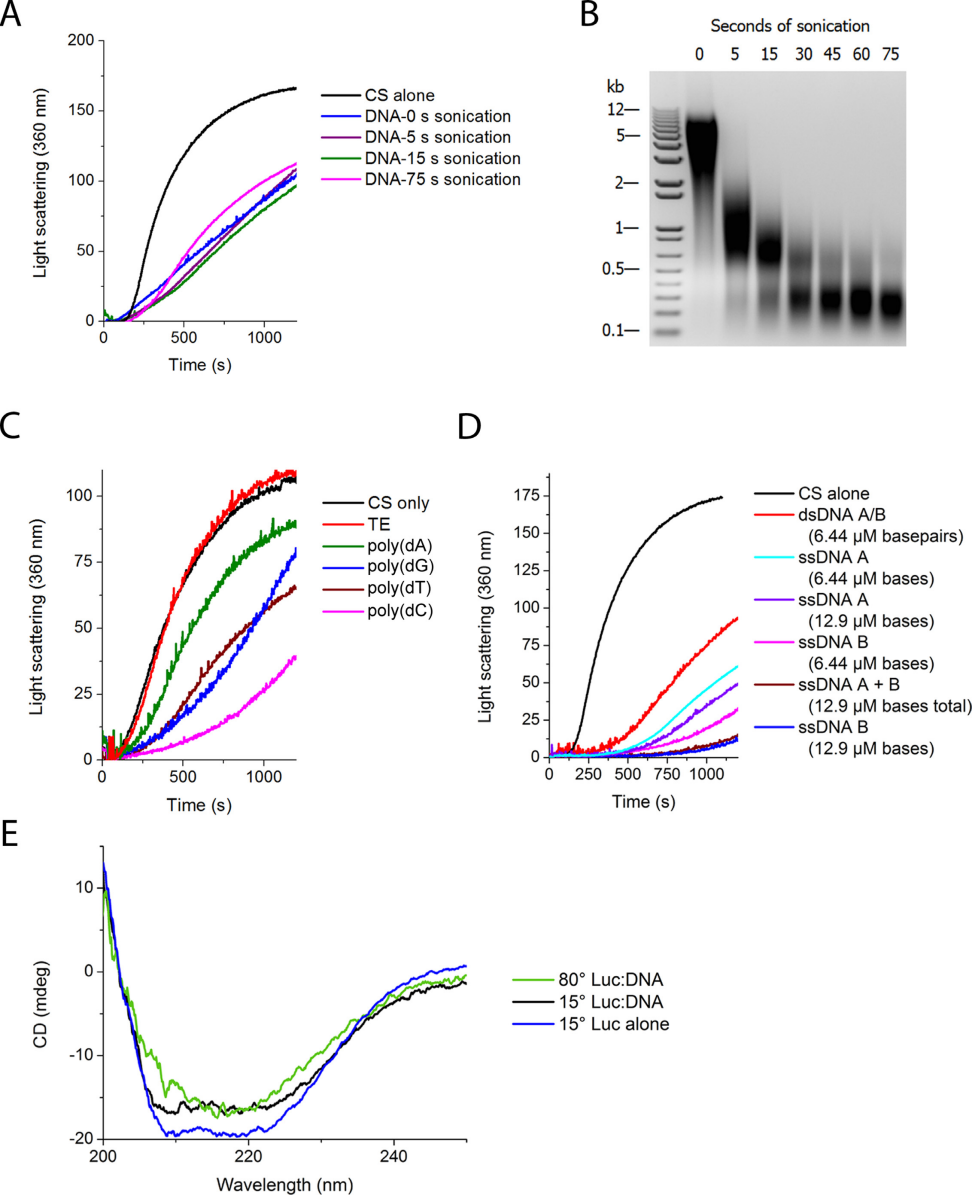
Protein aggregation in the presence of various nucleic acids. All were tested against thermal denaturation of 150 nM CS in HEPES buffer at 41°C. (**A** and **B**) Fragmented DNA. [DNA] = 322 nM basepairs. (**B**) One percent agarose gel of DNA samples used in (A). (**C**) Synthesized ssDNA 20 base-long oligos of indicated sequence. Oligos had been resuspended in Tris-EDTA (TE) buffer, which is included as a control. Final concentration was 1 μM bases. (**D**) Synthesized ssDNA and dsDNA of defined sequences (see ‘Materials and Methods’) 18 nucleotides in length. A/B indicates preannealed DNA to form double-stranded oligos, while A + B indicates both ssDNA A and ssDNA B were added to the reaction without preannealing. (**E**) DNA binds misfolded state of luciferase at high temperature. When excess herring genomic DNA is added at low temperature, a small change in spectrum is observed, suggesting that some portion of the luciferase is binding the DNA in a non-native conformation at low temperature. Heating luciferase in the presence of DNA causes a larger shift in the CD spectrum of the luciferase (after subtracting DNA CD spectrum). This large shift corresponds to a complete loss of helicity, according to BestSel quantification. Luciferase alone at high temperature aggregates and produces no detectable CD signal.

The length dependence was further examined by looking at different DNA fragment sizes produced by sonication and timed DNase digestion. Sonication of the DNA produced fragments ranging in size from ∼200 bp to 10 kb, but no difference in chaperone activity against CS aggregation was detected (Figure 4A and B). To confirm that it is indeed the polymeric nature of the DNA molecule and not the nucleotides themselves that confer the observed chaperone activity, DNase was added to the luciferase spin-down assays. The addition of DNase, which can degrade the DNA down to di- and trinucleotides, caused the luciferase to move nearly completely to the insoluble fraction, indicating that the DNA must be more than three nucleotides long to display chaperone activity (Figure 3C and D). Using timed DNase digests, we produced smaller fragments than sonication, and observed a clear negative correlation between chaperone activity and time of DNase digestion (Supplementary Figure S6). We then tested synthesized 30 base long random mixed sequences of ssDNA, as well as 18 and 71 base ssDNA oligomers of defined sequences, which all displayed chaperone activity roughly comparable to genomic DNA (Figure 2A and B). To test the length dependence more extensively, we synthesized random ssDNA nucleotides that varied in length between 15 and 60 bases. In contrast to the 18 base oligo (Figure 2A), which showed strong chaperone activity, the 15 base random oligos (Supplementary Figure S5) showed almost no chaperone activity, suggesting that the minimal length oligo for chaperone activity is somewhere around 15 to 18 bases. Longer oligos showed significant chaperone activity, though in a way that was not directly related to their length (Supplementary Figure S5). Although osmolytes have previously been shown to influence protein aggregation and refolding, they are only effective at much higher concentrations than nucleic acids. For instance, one of the most effective osmolytes, sucrose, must be included at concentrations of 100 mM to inhibit aggregation. By mass, this concentration is 300 000-fold higher than the quantity of RNA we found to exert a comparable effect upon aggregation (Figure 2C).

To determine if the sequence or strandedness of DNA affect chaperone activity, we tested several homopolymers and oligomers of ssDNA. Poly(dA) (20 base oligos) exhibited minimal effects on CS aggregation, whereas the same amount of poly(dC) reduced aggregation by 60% after 20 min. Poly(dG) and poly(dT) showed intermediate effects on aggregation, but with dissimilar kinetics (Figure 4C). In addition to sequence, strandedness appears to play a major role in chaperone activity. ssDNA oligos are more than twice as potent as their double-stranded counterparts, fragment for fragment, even though half the number of nucleotide residues are present (Figure 3D).

To evaluate if the anti-aggregation activity we observed stems primarily from nucleic acids binding to misfolded protein states, as would be expected of chaperones, we performed temperature-dependent CD measurements. In these experiments, genomic DNA and luciferase were incubated at low temperatures either separately or together. The temperature of the sample in the CD spectrometer was slowly increased. Consistent with the spin-down assay, luciferase by itself aggregated and precipitated with increased temperature, producing an undetectable CD signal. However, in the presence of DNA, the luciferase stayed in solution and a gradual structural change in the luciferase was observed (Figure 4E). This change in luciferase's CD spectra strongly suggests that the DNA binds a misfolded luciferase conformation at elevated temperature, as opposed to simply binding to and stabilizing the native conformation.

**Figure 5.**
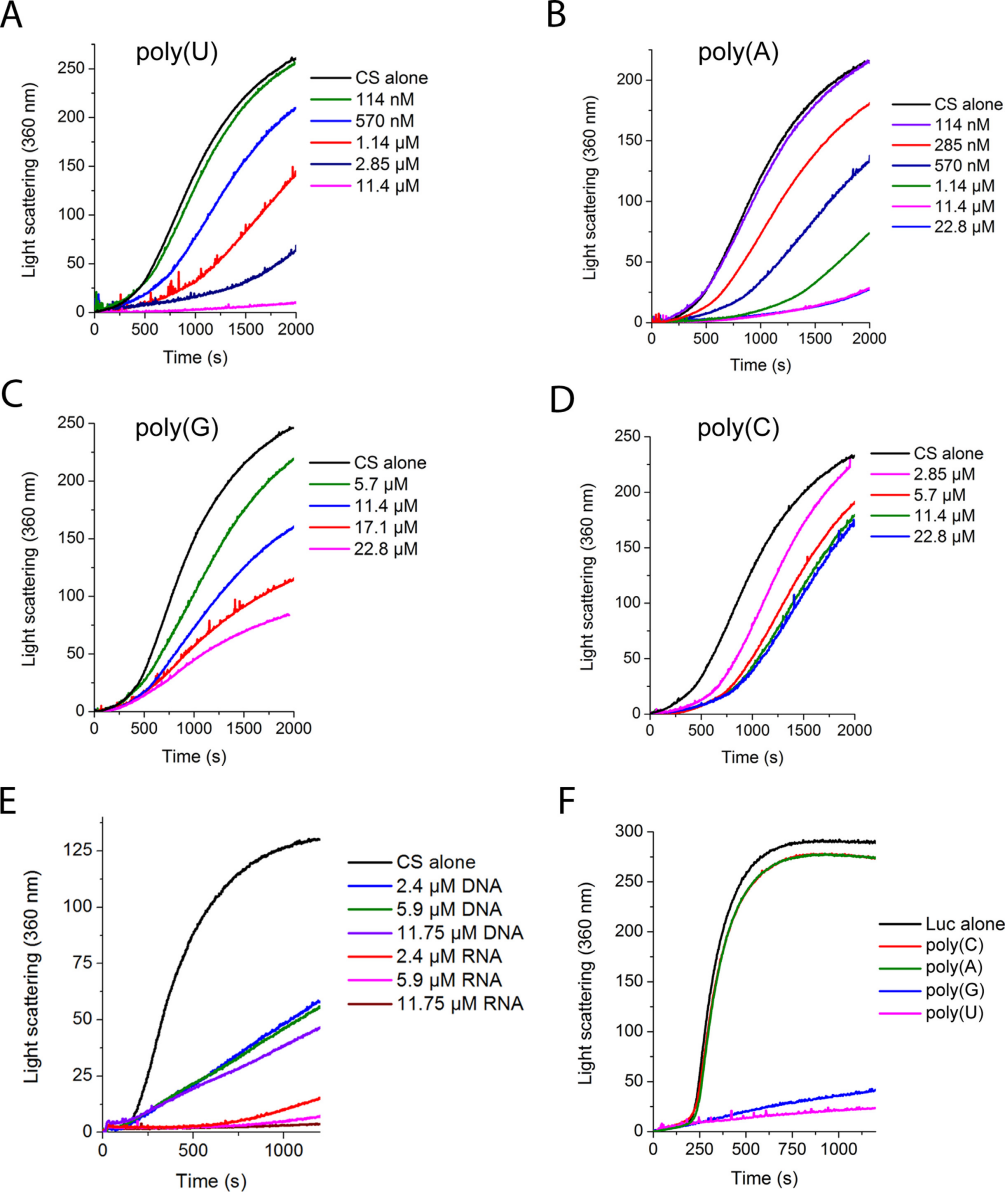
Protein aggregation in the presence of RNA. (**A–E**) Thermal denaturation of 150 nM CS in HEPES buffer at 41°C. Synthesized RNA oligos of 19 bases long consisting of (**A**) poly(U), (**B**) poly(A), (**C**) poly(G), or (**D**) poly(C) sequences; concentrations are per base. (**E**) Genomic herring DNA and bulk yeast RNA. Although the DNA is double-stranded, all concentrations are per base to facilitate comparison. (**F**) Synthesized RNA oligos of 19 bases long were tested against the thermal aggregation of 140 nM luciferase; RNA concentrations are 28.5 μM bases.

As ssDNA demonstrated ∼4-fold greater activity on a weight basis than dsDNA, we tested if RNA also has chaperone activity, using bulk RNA from yeast. RNA reduced the thermal aggregation of CS to an even greater extent than DNA, showing ∼5-fold higher chaperone activity (Figure 5E). Only 16 bases of RNA per CS molecule were required to reduce the rate of aggregation by ∼90%. Synthesized single stranded homopolymer RNA molecules also strongly inhibited CS and luciferase aggregation (Figure 5A–D). The most effective of these was poly(U). Poly(A) showed stronger activity than poly(U) at lower concentrations, but could not match the effectiveness of polyU at higher concentrations (Figure 5A and B). Poly(G) was ∼15-fold less effective than either Poly(A) or Poly(U) at all concentrations, but still had a significant effect (Figure 5C). Poly(C) was the least effective, showing even poorer activity than bulk DNA, and plateaued in activity before even reducing aggregation by 50% (Figure 5D). This sequence-specific activity was substrate dependent, as poly(U) and poly(G) showed strong activity against luciferase, whereas poly(A) and poly(C) showed little to no activity (Figure 5F).

**Figure 6.**
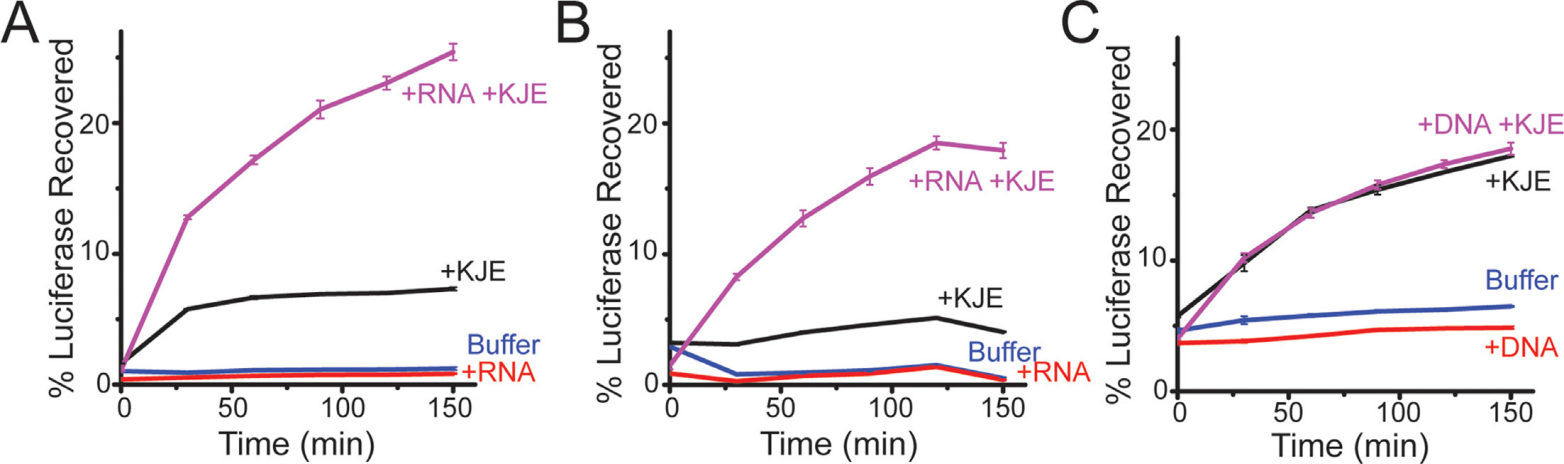
Luciferase refolding in the presence of nucleic acids. (**A** and **B**) 15 μM (per base) 19-base poly(U) RNA aids protein refolding by DnaK, DnaJ, GrpE (KJE) refolding system after heat inactivation at (**A**) 41°C or (**B**) 42°C. (**C**) Around 15 μM (per basepair) genomic herring DNA has no effect on luciferase refolding after heat inactivation at 40°C.

In addition to preventing aggregation, chaperones typically aid protein folding, either by directly enhancing protein folding or by transferring misfolded proteins to ATP-dependent chaperones for refolding. To examine whether nucleic acids can participate in protein refolding, we measured the recovery of luciferase activity after heat inactivation in the presence and absence of nucleic acids. Similar to small heat shock proteins (24,29,30), poly(U) RNA successfully transferred bound inactive luciferase to the DnaK chaperone system for refolding (Figure 6A and B). In contrast, DNA displayed little to no ability to transfer misfolded luciferase to the DnaK refolding system (Figure 6C). RNA is thus substantially more potent than DNA in both preventing aggregation and in transferring substrates to the DnaK system for refolding.

## DISCUSSION

As reviewed previously (31,32), there have been hints that nucleic acids may display chaperone activity toward proteins (4,33–36). These reports describe the effects nucleic acids have on the folding of nucleic acid-binding proteins, and it is well known that the conformational stability of a protein increases by binding its ligands (37). It has previously been shown that domain V of 23S rRNA is capable of refolding proteins *in vitro* (8–10,12,33,38), and nucleic acids can alter the folding of amyloid-forming proteins, leading to their misfolding and aggregation (39–44).

In this work, we demonstrate that a wide array of nucleic acids serve as extremely efficient anti-aggregation chaperones. Our results suggest that nucleic acids stabilize proteins by binding misfolded or partially folded protein states. We also show RNA can cooperate with the DnaK to facilitate protein refolding, in this way acting similarly to small heat shock proteins (24,29,30).

Nucleic acids’ general chaperone activity is consistent with recent investigations into the role of mRNA in stress granules. Stress granules form under conditions that lead to the inhibition of translation such as heat and oxidative stress (45,46). Stress granules comprise a heterogenous mixture of biological molecules, especially mRNAs, RNA-binding proteins, translation initiation factors, chaperones such as Hsp70 and CCT components, and proteins with predicted prion-like domains (47). During stress, the cell disrupts polysomes to halt translation, leading to a spike in free mRNAs, which then form stress granules (48,49). Stress granules were previously considered important as mRNA storage or degradation complexes, but this hypothesis has recently come under question (49–51). Recently it has been hypothesized that these mRNAs freed by translation inhibition act to recruit aggregation and amyloid-prone proteins to stress granules, which then protect these proteins in times of stress (52). Given our demonstration that a wide variety of nucleic acids have potent chaperone activity *in vitro*, we speculate that one possible *in vivo* role of these stress granules may be in protein folding, where mRNA cooperates with chaperones such as Hsp70 to facilitate protein refolding, or at a minimum, to keep proteins relatively soluble until stress is relieved. Establishing the *in vivo* importance of nucleic acids as chaperones is, however, rather difficult compared to genetically establishing the importance of individual proteins, as generating a nucleic acid-free organism presents a number of experimental and conceptual challenges.

One can imagine possible commercial applications for nucleic acids in protein stabilization or refolding. Although these applications will clearly require additional experimentation, one advantage of note is that purified DNA is about five orders of magnitude less expensive than purified GroEL.

Nucleic acids are such potent chaperones that if a preparation of a known protein chaperone such as GroEL contained even 1% RNA contamination by weight, the majority of the anti-aggregation activity observed against substrates such as CS would be due to the nucleic acid, not the GroEL. Moreover, the total concentration of nucleic acids in *E. coli* is around 300 mM in base-pair equivalents, which is about six orders of magnitude higher than the nucleic acid concentrations that showed positive chaperone activity in our *in vitro* assays (53). If nucleic acids are as potent *in vivo* as they are *in vitro*, then only a small percentage of nucleic acids would need to be available for the bulk of the anti-aggregation chaperone activity in the cell to be caused by nucleic acids, as opposed to proteins. The high potency we demonstrate here, combined with the ubiquity of cellular RNA, opens up the possibility that nucleic acids are biologically important chaperones.

The mechanism behind the chaperone activity of nucleic acids or other polyanions such as polyphosphate is not clear. Interestingly, although nucleic acids and polyphosphate act upon similar substrates, nucleic acids are much more efficient. We speculate that this increased activity could stem from nucleobases binding exposed hydrophobic patches and shielding them from aggregation. Furthermore, particular secondary structure elements in RNA may modulate chaperone activity. We found simple sequence variants of ssDNA and RNA to have a range of chaperone activities, pointing to a sequence, structural, or dynamic dependence for chaperone activity.

Protein–protein interactions and protein–solvent interactions are primarily what people consider when thinking about protein folding and aggregation. Our findings suggest we may need to expand the universe of cellular macromolecules that globally affect protein folding and aggregation to include nucleic acids.

## Supplementary Material

Supplementary Data

SUPPLEMENTARY DATA
