# Supplementary material for: Do nucleic acids moonlight as molecular chaperones?
Source: Nucleic Acids Res. 2016 Apr 21;44(10):4835–45. doi: 10.1093/nar/gkw291 (PMC4889950; doi:10.1093/nar/gkw291)
Supplement: Supplementary Data [file gkw291_Supplementary_Data.zip › nar-00594-h-2016-File008.pdf]

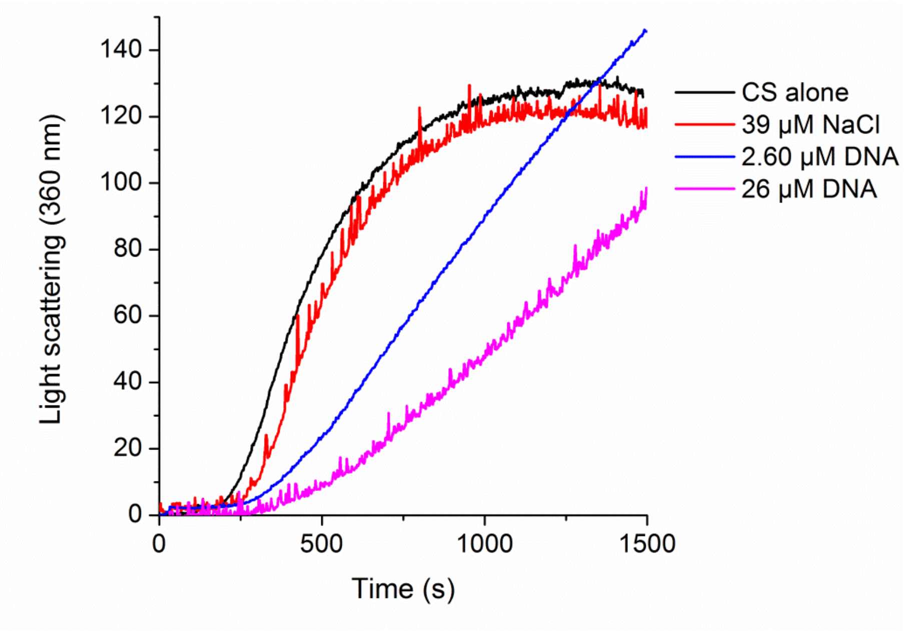

Figure S1: Effect of DNA on citrate synthase aggregation in Tris buffer. Thermally-induced aggregation of citrate synthase (CS) in 50 mM Tris, pH 8.0. Genomic herring DNA was used, and concentration is per base pair. [CS]=150 nM.

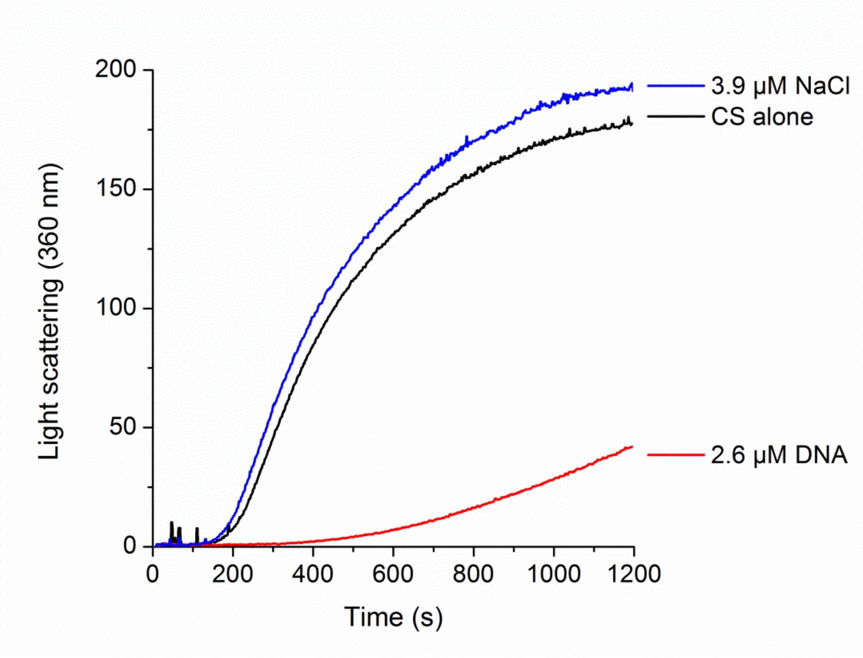

Figure S2: Effect of DNA on citrate synthase aggregation in MOPS buffer. Thermally-induced aggregation of citrate synthase (CS) in 50 mM MOPS, pH 7.5. Genomic herring DNA was used, and concentration is per base pair. [CS]=150 nM.

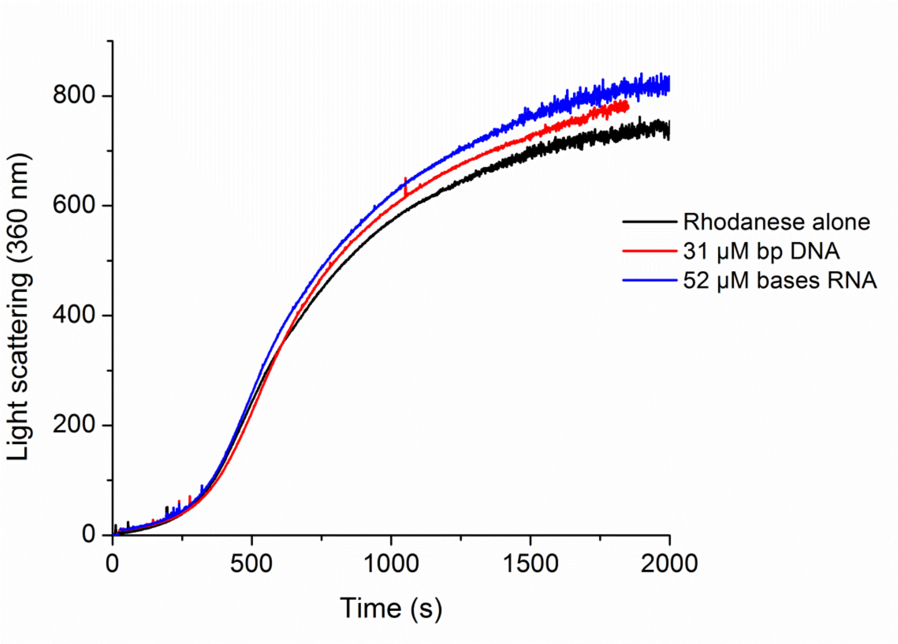

Figure S3: Effect of DNA on rhodanese thermal aggregation. Thermally-induced aggregation of rhodanese in the presence of bulk RNA from yeast or genomic herring DNA. [Rhodanese]=1.5 µM.

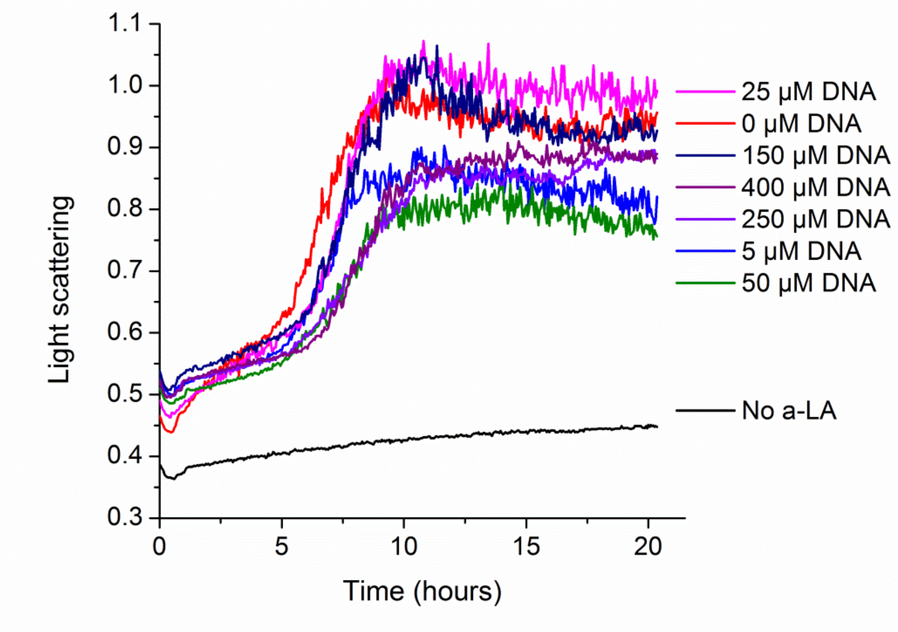

Figure S4: Effect of DNA on α-lactalbumin aggregation. Aggregation of α-lactalbumin (a-LA) in the presence of genomic herring DNA. DNA concentration is per base pair. [a-LA] =50 μM. Curves are an average of 3 technical replicates.

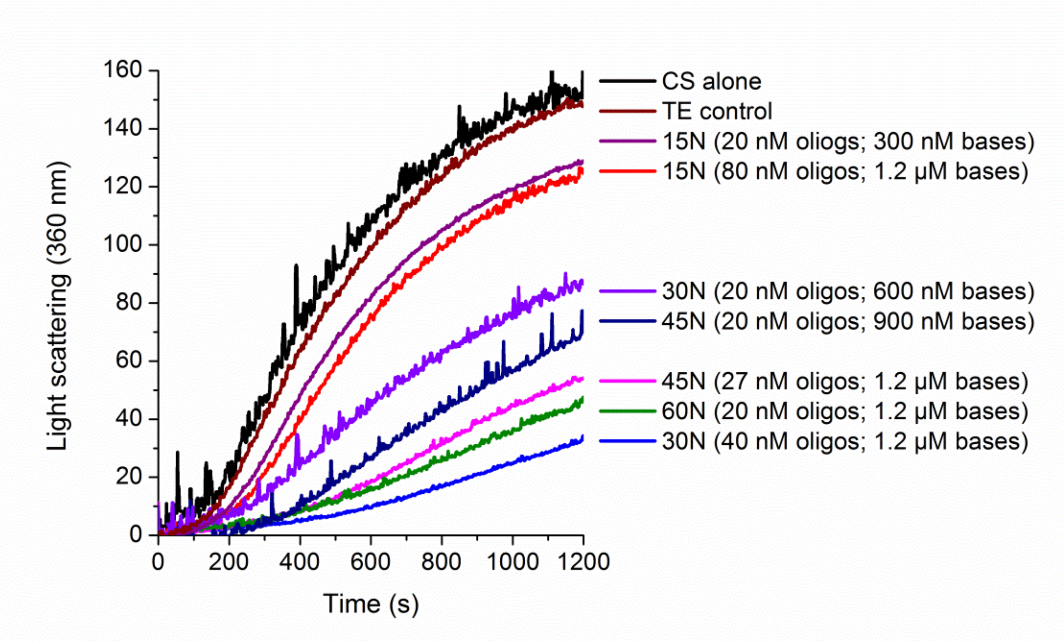

Figure S5: Effect of oligo length on aggregation. Thermally-induced aggregation of citrate synthase in the presence of synthesized ssDNA oligos of indicated lengths (e.g. 15N = 15 nucleotides in length). Oligos were a mix of random sequences. TE is the buffer control. [CS]=150 nM.

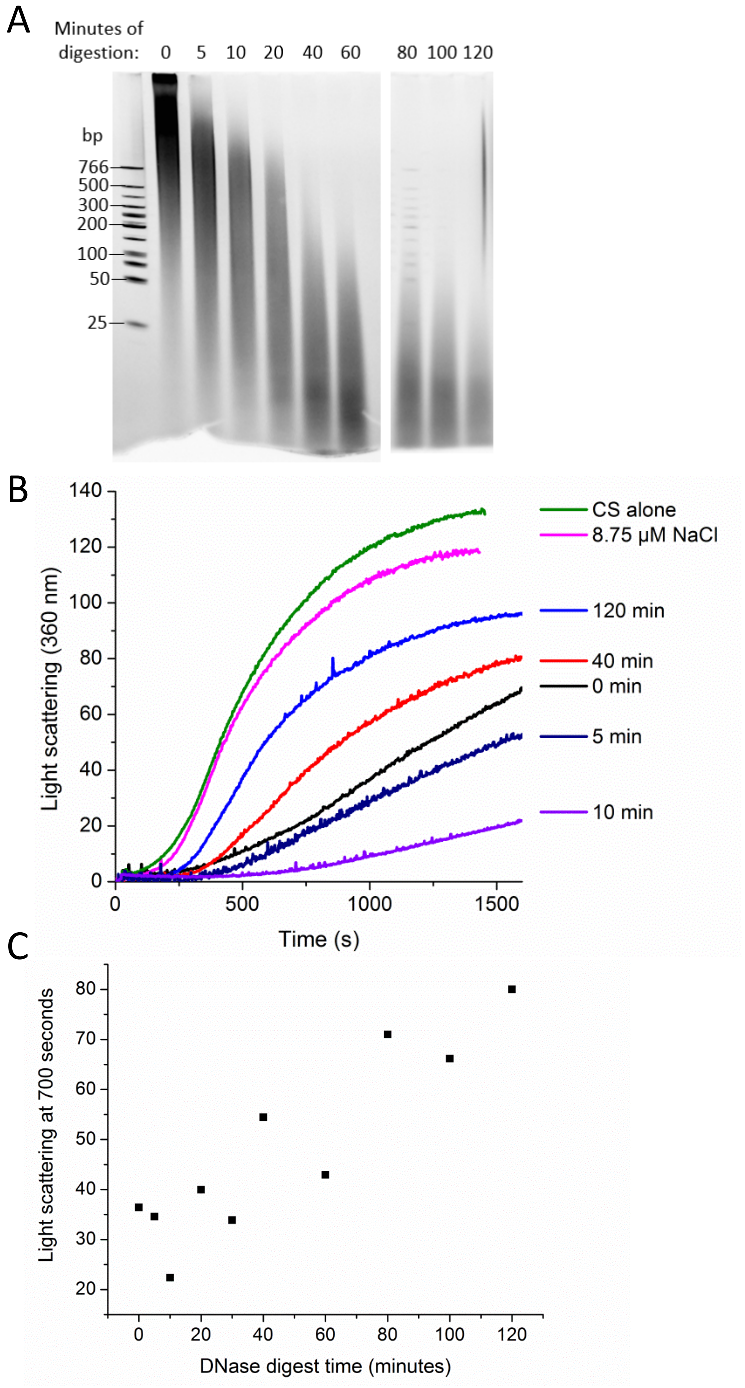

Figure S6: Effect of DNA digestion on its chaperone activity. DNA was digested for various amounts of time and tested for chaperone activity. (**A**) Size of DNA samples on a 20% polyacrylamide-TBE gel. Time indicates length of DNase digest of each DNA sample. Reaction contained 1.16 mM bp DNA, 20.2 ng/mL DNase in 50 mM Tris, 5 mM MgCl2, 130 μM CaCl2 at 26°C for indicated amount of time. (**B**) Thermally-induced aggregation of citrate synthase (CS) in the presence of the samples shown in **A**. For clarity, not all samples tested are shown. [DNA]=580 nM bp; [CS]=150 nM. (**C**) Signal level at 700 seconds in **B** relative to DNase digest time; lower scattering signal indicates higher chaperone activity.

Rhodanese (type II from bovine liver, Sigma-Aldrich) at 1.5 μM was incubated at 40°C in 40 mM potassium phosphate, pH 7.5, with constant stirring. Aggregation of 50 μM α-lactalbumin (from bovine milk, Sigma-Aldrich) in 50 mM sodium phosphate, 100 mM potassium chloride, 18 mM DTT, pH 7.0 was measured in a plate reader at 37°C, with absorbance at 360 nm measurements taken every 3 minutes with 10 seconds of shaking before each measurement.
