# Supplementary material for: Do nucleic acids moonlight as molecular chaperones?
Source: Nucleic Acids Res. 2016 Apr 21;44(10):4835–45. doi: 10.1093/nar/gkw291 (PMC4889950; doi:10.1093/nar/gkw291)
Supplement: SUPPLEMENTARY DATA [file supp_44_10_4835__index.html]

Do nucleic acids moonlight as molecular chaperones? — Do nucleic acids moonlight as molecular chaperones? — SUPPLEMENTARY DATA 

# Do nucleic acids moonlight as molecular chaperones?

## SUPPLEMENTARY DATA

- SUPPLEMENTARY DATA
